# Predictors of Hungry Bone Syndrome After Parathyroidectomy in Secondary Hyperparathyroidism: A Narrative Review of Bone Turnover Biomarkers and Risk Prediction Tools

**DOI:** 10.3390/jcm14217849

**Published:** 2025-11-05

**Authors:** Adina Coman, Cristi Tarta, Alexandru Isaic, Marco Marian, Sorin Olariu, Andrei Ardelean, Anca-Monica Macovei-Oprescu, Fazakas Roland, Gheorghe-Nanu Pupca, Silviu Latcu, Cristian Silviu Suciu, Marius Murariu

**Affiliations:** 1 Researching Future Surgery II Research Center, Department X, Discipline of General Surgery II, Faculty of Medicine, Victor Babes University of Medicine and Pharmacy Timisoara, E. Murgu Square, No. 2, 300041 Timisoara, Romania; adina.coman@umft.ro (A.C.); isaic.alexandru@umft.ro (A.I.); marian.marco@umft.ro (M.M.); 2 Doctoral School, Victor Babes University of Medicine and Pharmacy Timisoara, E. Murgu Square, No. 2, 300041 Timisoara, Romania; 3 Department X, Discipline of General Surgery I, Faculty of Medicine, Victor Babes University of Medicine and Pharmacy Timisoara, E. Murgu Square, No. 2, 300041 Timisoara, Romania; olariu.sorin@umft.ro (S.O.); murariu.marius@umft.ro (M.M.); 4 Department of Medicine, Discipline of Surgery I, Vasile Goldiş Western University, Liviu Rebreanu Boulevard, No. 86, 310414 Arad, Romania; ardelean.andrei@uvvg.ro; 5 Department V, Discipline of Internal Medicine II and Gastroenterology, Faculty of Medicine, University of Medicine and Pharmacy Carol Davila Bucharest, Eroii Sanitari Bvd., No. 8, Sector 5, 020021 Bucharest, Romania; anca.macovei@umfcd.ro; 6 Doctoral School, Faculty of Medicine, Vasile Goldiş Western University, Liviu Rebreanu Boulevard, No. 86, 310414 Arad, Romania; 7 Department XV, Discipline of Urology, Victor Babes University of Medicine and Pharmacy Timisoara, E. Murgu Square, Nr. 2, 300041 Timisoara, Romania; gheorghe.pupca@umft.ro (G.-N.P.); silviu.latcu@umft.ro (S.L.); 8 Department II of Microscopic Morphology, Victor Babes University of Medicine and Pharmacy Timisoara, E. Murgu Square, No. 2, 300041 Timisoara, Romania; cristi.suciu@umft.ro

**Keywords:** endocrine surgery, parathyroid, parathyroidectomy, hungry bone syndrome biomarkers, secondary hyperparathyroidism, bone turnover markers, alkaline phosphatase, chronic kidney disease, hypocalcemia, risk prediction models

## Abstract

**Background/Objectives**: Secondary hyperparathyroidism (SHPT) affects 30–50% of end-stage renal disease patients. Parathyroidectomy (PTX), while effective for medication-refractory SHPT, carries 20–70% risk of hungry bone syndrome (HBS)—severe sustained hypocalcemia requiring intensive care and prolonged hospitalization. Accurate preoperative risk stratification using biochemical markers and validated prediction tools is critical for optimal preventive management. **Methods**: We conducted a comprehensive narrative review synthesizing evidence on HBS predictors after PTX in SHPT, evaluating traditional and novel bone turnover markers, clinical risk factors, and multivariate prediction models, through a structured literature search and analysis. **Results**: Preoperative bone turnover status represents the strongest contributor to HBS risk. Traditional biomarkers—particularly parathyroid hormone (PTH > 1000–2400 pg/mL) and alkaline phosphatase (ALP > 150–300 U/L)—demonstrate moderate-to-strong individual predictive power. Novel bone turnover markers (bone-specific ALP, P1NP, TRAP-5b) offer incremental value, especially in CKD populations where renal clearance affects traditional markers. Combined risk prediction models substantially outperform single biomarkers, achieving area under curve values of 0.87–0.95. The simple NYU 2-point score (ALP > 150 U/L + PTH > 1000 pg/mL) showed 96.8% accuracy, with 100% negative predictive value. More complex tools like nomograms (C-index 0.92–0.94) and machine-learning algorithms (AUC 0.88) provide enhanced discrimination by integrating multiple continuous parameters. Additional clinical factors—younger age (<48 years), prolonged dialysis (≥5 years), low preoperative calcium, high gland weight, and absence of autotransplantation—further refine risk assessment. Postoperative calcium typically reaches nadir at 48–72 h, defining the critical monitoring window. **Conclusions**: High-turnover bone biomarkers and combined risk models effectively identify high-risk SHPT patients. Risk-stratified protocols (i.e., prophylactic supplementation, intensive monitoring, and selective ICU admission) can substantially reduce HBS-related morbidity. Ongoing efforts should focus on validating these predictive tools across diverse populations and integrating them into clinical practice, thereby facilitating real-time HBS risk assessment and protocol-driven care.

## 1. Introduction

Secondary hyperparathyroidism (SHPT) represents a significant complication of chronic kidney disease (CKD), affecting 30–50% of end-stage renal disease (ESRD) patients and contributing substantially to morbidity and mortality [1,2]. In SHPT, disrupted mineral metabolism (phosphate retention, vitamin D deficiency, and hypocalcemia due to renal failure) triggers compensatory parathyroid hyperplasia and excessive parathyroid hormone (PTH) release, driving abnormally high bone turnover. This high-turnover renal osteodystrophy (historically osteitis fibrosa cystica) involves accelerated endosteal and intracortical bone resorption with cortical thinning, reduced overall bone mass (cortical bone represents ~75% of this total), and defective mineralization, with reduced bone mineral density (BMD) [3]. Thus, as kidney function declines, adaptive responses become maladaptive, ultimately manifesting as severe skeletal and cardiovascular complications characteristic of CKD-mineral and bone disorder (CKD-MBD) [4].

While medical management with phosphate binders, calcitriol analogs, and calcimimetics remains the first-line therapy, parathyroidectomy (PTX) becomes necessary in ~15% of ESRD patients after 5–10 years of ongoing dialysis (versus 38% after 20 years), due to the onset of medication-refractory SHPT [5]. Indications for surgical intervention include persistently elevated PTH levels (>800–1000 pg/mL), with hypercalcemia and/or hyperphosphatemia, despite optimal medical therapy, as well as calciphylaxis, pruritus, severe bone pain/fractures, and/or progressive extraskeletal calcification [6]. Herein, PTX is effective not only in reducing PTH levels but also in improving biochemical profiles overall and patient survival, decreasing erythropoietin resistance, increasing BMD, and reducing the risk of fractures in dialysis patients with SHPT. However, PTX carries an inherent risk of hungry bone syndrome (HBS), a severe metabolic complication characterized by a potentially life-threatening persistent hypocalcemia [7].

HBS is caused by acute, aggressive postoperative bone remineralization, following the abrupt cessation of PTH-mediated bone resorption, while osteoblastic bone formation continues unopposed. This process creates a massive calcium and phosphate demand that outstrips the body’s compensatory capacity [7]. The acute phase typically begins within 12–24 h postop, with calcium levels reaching their nadir between days 2–3 (up to 7), often accompanied by hypophosphatemia and hypomagnesemia [7]. Thereafter, the consequences of HBS may extend far beyond simple electrolyte imbalance, with clinical manifestations ranging from mild perioral tingling and muscle cramps, to more important complications including tetany, seizures, cardiac arrhythmias, aggravated congestive heart failure and laryngospasm. These cases may necessitate prolonged hospitalization, intensive care unit (ICU) admission, and massive intravenous calcium supplementation [8]. Moreover, protracted HBS courses lasting weeks to months mandate careful long-term monitoring and management, which, alongside potential readmissions, create ongoing financial strain within healthcare systems already burdened by the high costs of ESRD care [9]. Thus, accurate preoperative risk stratification is crucial to guide targeted preventive measures and optimal resource allocation.

Reported HBS incidence post-PTX in SHPT varies widely (~10–92%) [7,10], somewhat mirroring, albeit to a lesser extent, the heterogeneity observed in primary (P)HPT. However, SHPT cases usually exhibit consistently higher HBS rates (often >50%) overall, versus primary cases (~13–25%) [11,12]. This wide variation reflects underlying differences in diagnostic criteria, patient populations, surgical techniques, and pre-op management strategies. For instance, using a strict cutoff (e.g., nadir calcium < 1.88 mmol/L or <7.5 mg/dL) yields lower incidence (~20–30%), whereas more inclusive criteria (any prolonged hypocalcemia requiring supplementation) find HBS in the majority of dialysis patients.

Even so, a recent systematic review analyzing >2500 patients outlined that SHPT patients face a 43.6% HBS risk, as compared to only 15.9% in primary and 12.4% in tertiary HPT, respectively, with significant implications for healthcare resource allocation [12]. It is well recognized that patients with more severe underlying bone disease carry the highest risk. Therefore, bone turnover markers (BTMs) provide a window into the dynamic processes of bone remodeling, offering both pathophysiological insights and potential practical tools for preoperative HBS risk assessment.

Currently, PTH is the most common index used to evaluate the severity of CKD-MBD. With the gradual decline of renal function in ESKD patients, the phosphate scavenging effect of the renal tubule also decreases accordingly; meanwhile, inadequate vitamin D activation occurs to maintain the normal serum calcium. Thus, excessive PTH disrupts the balance between bone formation and resorption, driving high-turnover bone disease [13,14,15].

Beyond PTH, several biomarkers reflect different aspects of bone metabolism relevant to HBS risk. Alkaline phosphatase (ALP) activity is crucial for proper bone mineralization and is a well-recognized biomarker of renal osteodystrophy [4,16,17]. In SHPT, serum ALP levels rise with osteoblast activation, and the magnitude of this increase correlates with the severity of bone disease [18,19,20]. Preoperative ALP concentrations tend to be higher in patients who develop HBS, reflecting more active bone remodeling in these individuals [12,21]. The repertoire of HBS prediction tools has expanded, from traditional markers such as total ALP and PTH, to include newer BTMs like bone-specific ALP (BALP), tartrate-resistant acid phosphatase 5b (TRAP-5b), C-Terminal and N-Terminal Telopeptides (CTX/NTX), and procollagen type I N-terminal propeptide (P1NP), as well as regulatory molecules such as fibroblast growth factor-23 (FGF-23) and sclerostin, all currently under investigation [22,23,24]. Each marker provides unique information about specific aspects of bone metabolism, with varying utility in the CKD population due to factors such as renal clearance, assay standardization, and biological variability.

In this comprehensive narrative review, we synthesize current molecular pathology knowledge on HBS in the context of SHPT and the utility of BTMs for predicting this complication. We examine both traditional and novel BTMs (including ALP, BALP, PTH, P1NP, TRAP-5b, CTX/NTX, FGF-23, and others) in relation to HBS risk stratification. Furthermore, we discuss emerging predictive models and risk assessment tools, highlighting their potential to inform clinical decision-making in the perioperative setting.

Finally, we emphasize the translational relevance of predictive modeling for HBS risk. Effective risk stratification tools could enable clinicians to implement proactive measures—preoperative calcium supplementation protocols or closer postoperative monitoring—in patients identified as high risk, thereby mitigating severe hypocalcemic complications. In addition, predictive models can guide resource allocation by distinguishing which patients may require intensive care or prolonged hospitalization. By integrating such biomarker-driven predictive strategies into practice, patient outcomes could be improved and the healthcare burden associated with HBS in the dialysis population reduced.

This comprehensive narrative review was informed by a systematic PubMed/MEDLINE database search (latest update October 2025) for English-language articles using the keywords “hungry bone syndrome,” “secondary hyperparathyroidism,” “parathyroidectomy,” “bone turnover markers,” “alkaline phosphatase,” “parathyroid hormone,” “chronic kidney disease,” “hypocalcemia,” and “risk prediction models.” We identified and screened relevant original research articles, meta-analyses, systematic reviews, clinical practice guidelines, and seminal foundational studies. Priority was given to contemporary evidence published within the last decade, including large cohort studies, validation studies of prediction models, and recent systematic reviews, while also incorporating landmark earlier works to provide foundational mechanistic and physiological context. Studies evaluating both traditional biomarkers (PTH, ALP) and novel markers (BALP, P1NP, TRAP-5b, CTX, FGF-23, sclerostin) were included, as well as investigations of multivariate risk scores and machine-learning (ML) prediction tools. Reference lists of key articles were manually reviewed to identify additional relevant studies. The evidence was synthesized qualitatively to highlight areas of consensus, identify knowledge gaps and conflicting findings, compare predictive performance across different biomarkers and models, and provide an integrated overview of HBS pathophysiology, risk stratification strategies, and perioperative management protocols.

## 2. Parathyroid Hormone Biology and Pathogenesis

### 2.1. Metabolism and Receptor Signaling

PTH is a polypeptide critical for calcium and phosphate homeostasis [25]. It is translated in the parathyroid chief cells as a 115–aminoacid preprohormone, which is cleaved to a 90–aminoacid pro-PTH, and then to the bioactive 84–aminoacid form—intact (i)PTH—which is then stored in secretory granules [26]. The circadian pulsatile secretion of PTH is tightly regulated by the calcium-sensing receptor (CaSR) on the surface of parathyroid cells, which detects minute changes in extracellular ionized calcium [25]. Hypocalcemia triggers a rapid increase in PTH release (within minutes), while hypercalcemia activates CaSR to suppress PTH secretion and synthesis at the gene level [25].

Phosphate and vitamin D also modulate PTH levels: hyperphosphatemia (even independent of calcium changes) stimulates PTH secretion, partly via a direct inhibitory effect of high phosphate on CaSR [27]; whereas 1,25-dihydroxyvitamin D (calcitriol) acts on vitamin D receptors (VDRs) in parathyroid cells to repress PTH gene transcription [25]. Likewise, FGF-23, a phosphaturic hormone from bone, binds the FGF receptor/Klotho complex on parathyroid cells to decrease PTH synthesis [28,29]. Under normal physiology, low calcium, high phosphate, and low calcitriol drive PTH up, whereas elevated calcium, calcitriol, and FGF23 impose a brake on PTH release [30].

Once secreted, iPTH(1–84) has a short plasma half-life of only ~2–4 min [31]. It is rapidly metabolized, mainly by the liver and kidneys, into N-terminal and C-terminal fragments, respectively, [32]. The N-terminal (1–34) fragment retains full bioactivity, whereas C-terminal fragments are generally inert at the classic type-1 PTH/PTHrP (related peptide) receptor—PTH1R [31,32]. In normal physiology, the kidneys efficiently clear these PTH fragments; however, advanced renal failure impairs fragment clearance, leading to accumulation of long-lived C-terminal species. Indeed, in CKD patients, up to ~50% of circulating PTH consists of such fragments [33]. Conversely, hypercalcemic conditions favor preferential production of C-terminal PTH fragments (which have blunted biological activity) as a feedback mechanism to reduce calcium elevation [33].

In addition to the PTH1R, recent evidence indicates the existence of an additional C-terminal PTH receptor (CPTHR), highly expressed on osteocytes in bone tissue, which distinctly binds PTH(7–84) fragments. Thus, even though C-terminal fragments do not activate the PTH1R, they may antagonize PTH1R-mediated actions and even elicit independent signaling via this emerging CPTHR [34]. In fact, it appears that PTH(7–84) can directly suppress osteoclastogenesis and bone resorption, both in vitro and in vivo, effects that are opposite to and independent of PTH1R, and thus presumably mediated by this novel C-fragment-specific variant [34]. Therefore, this “C-PTH” pathway may actually serve as a physiological counter-regulatory mechanism to temper PTH’s bone-resorptive effects. In renal failure, however, high circulating levels of PTH(7–84) and related peptides [33], acting via CPTHR, may in fact contribute to the skeletal resistance to PTH observed in uremia, by blunting the calcemic and resorptive effects of iPTH [34].

Even so, the central biological actions of PTH are exerted by binding to the PTH1R, a cell-surface, high-affinity, G-protein–coupled, class B/secretin-like receptor, abundantly expressed on cellular effectors in main target tissues, namely in bone and kidney, i.e., on osteoblasts and osteocytes, respectively, on renal tubular cells [35]. When PTH1R is activated by PTH (or the derived parathyroid hormone–related peptide, PTHrP), it primarily engages G_s_ and G_q/11_ heterotrimeric G-proteins, which stimulate two major signaling cascades: the adenylate cyclase–cyclic (c)AMP-protein kinase A (PKA) pathway and the phospholipase C/inositol trisphosphate–protein kinase C (PKC) pathway [36,37]. Through these pathways, PTH1R activation rapidly alters ion transport, gene expression and cellular behavior in its target effectors.

### 2.2. Parathyroid Hormone Cellular Effectors and Main Biological Effects in Bone Tissue

In healthy bone, PTH1R is expressed on osteoblastic-lineage cells—specifically osteoblasts and osteocytes—where it orchestrates a carefully balanced skeletal response. Herein, the biological actions of PTH are inherently dichotomous, exerting either anabolic (bone-building) or catabolic (bone-resorbing) effects depending critically on the pattern of hormone exposure [37].

Intermittent PTH exposure, as achieved with once-daily teriparatide administration, preferentially stimulates osteoblast activity and promotes bone formation. Upon pulsatile PTH1R signaling, several anabolic cascades are triggered: upregulation of pro-osteogenic genes (including *Runx2*, *Osterix*, and *Col1A1*) via the cAMP/PKA pathway [38], inhibition of osteoblast-lineage apoptosis (effectively prolonging osteoblast lifespan), and enhancement of Wnt/β-catenin signaling through suppression of sclerostin, a key inhibitor of this pathway [38,39]. In osteocytes specifically, PTH signaling rapidly downregulates sclerostin (the *SOST* gene product) via phosphorylation of transcription factors such as CREB and MEF2. This downregulation effectively “lifts the brakes” off Wnt signaling, thereby promoting osteoblastic differentiation and osteogenesis [40,41]. The cumulative result is increased osteoid production and mineralization, leading to net gains in bone mass. In corroboration of these molecular mechanisms, intermittent PTH exposure has been exploited therapeutically (PTH-analog administration in osteoporosis), having been documented to reduce osteocyte sclerostin expression, while simultaneously increasing bone mass [42].

In sharp contrast, sustained PTH elevation—as occurs in hyperparathyroid states—elicits catabolic dominance. Under chronic stimulation, PTH1R signaling in osteoblasts and osteocytes induces high levels of receptor activator of NF-κB ligand (RANKL), while suppressing osteoprotegerin (OPG), thus tipping the balance toward osteoclast differentiation and enhanced bone resorption [43,44,45]. Through this mechanism, PTH stimulates osteoblast-lineage cells to indirectly recruit and activate osteoclasts—PTH-driven RANKL from osteoblasts binds RANK on osteoclast myeloid precursors, promoting their maturation into active bone-resorbing cells [43,44]. Simultaneously, the reduction in OPG—which normally acts as a decoy receptor for RANKL—removes a critical inhibitory restraint on the RANKL–RANK interaction [45]. This RANKL/OPG-mediated pathway represents the fundamental mechanism by which PTH mobilizes calcium from skeletal stores.

Osteocytes—constituting approximately 95% of all bone cells—play a particularly active role under sustained PTH stimulation. Beyond increasing RANKL expression, they upregulate cytokines such as monocyte chemoattractant protein-1 (MCP-1) [44], which recruits pre-osteoclast cells from bone marrow and enhances their fusion into multinucleated osteoclasts. Supporting MCP-1’s mechanistic importance, blocking this chemokine in murine models protects against PTH-induced bone loss [46]. Additionally, chronically stimulated osteocytes engage in osteocytic osteolysis, directly resorbing the perilacunar bone matrix surrounding them [34].

**Figure 1:**
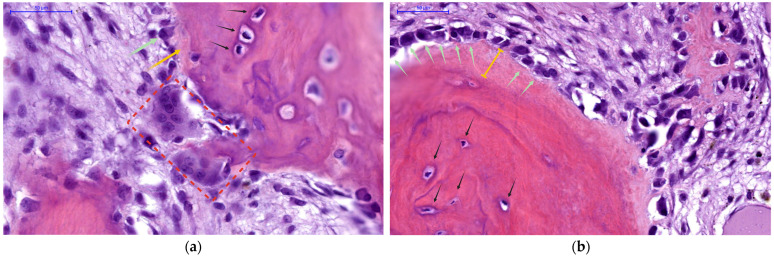
Histological details of human bone (400×, H&E stain), focusing on the endocortical surface to demonstrate parathyroid hormone (PTH) cellular effectors and duality of actions: (**a**) Active resorption—Multinucleated osteoclast (red rectangle) actively eroding bone matrix, visible as it further excavates within its resorption lacunae. Scarce surface osteoblasts (green arrow) can be seen depositing scant amounts of unmineralized osteoid (yellow arrow), while deeper in the matrix, a network of osteocytes (black arrows) resides within the mineralized bone. This panel depicts the catabolic phase of PTH action, during which calcium is mobilized to restore serum levels; (**b**) Enhanced woven bone formation—Expansive pool of unmineralized osteoid (yellow brackets), covers the bone surface, produced by numerous active osteoblasts (green arrows), lined up in a continuous layer. Beneath this osteoid layer lies the pre-existing mineralized bone containing mature embedded osteocytes (black arrows). In this panel, PTH’s anabolic action predominates, stimulating new (woven) bone matrix deposition and robust osteoblast activity. NB: Archival microscopic images from our Histology slide bank.

To fully appreciate the physiological basis of PTH’s biological effects in bone, it is essential to examine the histology of its primary cellular targets: osteoclasts, osteoblasts, and osteocytes. Each cell type plays a distinct yet coordinated role in bone remodeling under PTH signaling:

1. Osteoclasts are large, multinucleated cells responsible for bone resorption, as highlighted in Figure 1a (red rectangle). These cells are formed from myeloid precursors under the influence of osteoblast/osteocyte-derived RANKL [43,44,45]. Importantly, they do not express PTH1R and therefore are only indirectly responsive to PTH, with bone-resorptive actions being entirely mediated through osteoblast-lineage cells.
2. Osteoblasts, derived from MSCs, are responsible for forming new bone matrix (osteoid) and regulating its subsequent mineralization, as seen in Figure 1b (green arrows); they also orchestrate osteoclast activity by producing RANKL (and suppressing OPG) in response to sustained PTH signaling [43,44,45].
3. Osteocytes (marked with black arrows in Figure 1) represent former osteoblasts that have become embedded within the mineralized bone matrix. They function as endocrine cells within bone tissue, sensing both mechanical and hormonal signals, while also upregulating and secreting regulatory factors—most notably MCP-1 and sclerostin, respectively—which in turn modulate the activity of both osteoblasts and osteoclasts [43,44,45,46]. Osteocytes express PTH1R and are primary targets of PTH action.

PTH thus leverages these three cell types in concert to achieve bone remodeling: in tandem, it stimulates osteoblast-lineage cells either to build new bone or to recruit osteoclasts for bone resorption (as seen in Figure 1), striking a balance that is crucial for calcium homeostasis and skeletal integrity. However, these cellular mechanisms also represent the substrate for pathological high-turnover bone disease when PTH secretion becomes chronically elevated, as in SHPT.

To sum up, the net skeletal effect of PTH depends fundamentally on whether hormone exposure is intermittent or continuous, as illustrated histologically in Figure 1. Each panel demonstrates one side of the dual nature of PTH-driven bone remodeling: in areas of active resorption, osteoclasts dominate the landscape (Figure 1a); whereas in areas of vigorous new bone formation (Figure 1b), abundant osteoblasts produce expansive pools of unmineralized osteoid. The coexistence of enhanced bone breakdown and matrix accumulation represents the hallmark of PTH-driven skeletal dynamics, as seen in SHPT.

### 2.3. The Bone-Kidney Axis and Other Non-Canonical Effects in Normal Physiology

PTH is classically viewed as the chief regulator of calcium and phosphate balance through its coordinated actions in bone and kidney. In bone, as described above, PTH increases bone turnover to mobilize calcium. Acute or intermittent increases in PTH stimulate osteoblastic bone formation and can even improve bone microarchitecture, whereas sustained elevations in PTH trigger osteoblast-driven osteoclast activation and bone breakdown [37]. This bone-resorptive mechanism is critical for short-term calcium homeostasis. Notably, PTH also has a pro-survival effect on osteocytes, reducing their apoptosis and thereby preserving the mechano-sensory network in bone [47]. In essence, PTH’s skeletal effects constitute a tightly regulated remodeling exercise: transient PTH elevation favors osteoblastic osteogenesis, whereas chronic PTH excess leads to net bone loss due to unchecked osteoclast activity, which skews the balance toward bone resorption and calcium efflux from the skeleton [37].

Complementary to its central bone metabolism effects, PTH also acts on the kidneys to fine-tune mineral reabsorption and excretion. PTH1R is expressed in renal tubular cells, especially in the thick ascending limb and distal nephron. Upon PTH binding, the kidneys markedly increase calcium reabsorption in the distal tubules and collecting ducts, largely by upregulating apical calcium channels (TRPV5) and intracellular calcium-binding proteins (calbindin-D28k) within the aforementioned target segments [48]. At the same time, PTH has a phosphaturic effect: it inhibits phosphate reabsorption in the proximal tubule by triggering the internalization and lysosomal degradation of sodium-phosphate cotransporters (NaPi-2a/2c) in the epithelium lining this target segment, thereby increasing urinary phosphate excretion [49]. The net renal effect of PTH is to conserve urinary calcium (i.e., raising serum levels), while enhancing urinary phosphate excretion (i.e., lowering serum levels). PTH also stimulates the renal 1α-hydroxylase enzyme (CYP27B1) in proximal tubule cells, enhancing the conversion of 25-hydroxyvitamin D into active calcitriol, while also suppressing 24-hydroxylase (CYP24A1), the enzyme responsible for vitamin D catabolism [50]. The resulting rise in calcitriol amplifies intestinal calcium absorption via upregulation of TRPV6 channels, while also simultaneously providing negative feedback on the parathyroid glands (via parathyroid VDRs) to suppress further PTH release, thus completing the homeostatic endocrine loop [51]. In these ways, PTH orchestrates a multi-organ response to hypocalcemia: extracting calcium from bone, conserving urinary calcium (and dumping phosphate) in the kidney, and increasing intestinal calcium uptake via enhanced vitamin D activation.

PTH’s actions extend beyond the bone–kidney axis, reflecting its broader systemic effects. For instance, experimental murine studies suggest that pancreatic islets [52] may constitute an emerging PTH-target, which most likely associates with a still undescribed PTH receptor variant, modulating insulin release in a calcium- and cAMP-dependent manner [53]. Clinically, states of excess PTH secretion (e.g., P/SHPT) are associated with impaired insulin secretion and glucose intolerance, which improve after PTX—suggesting an occult, yet reversible, PTH-mediated effect on pancreatic islet function [53]. In the bone marrow, PTH influences MSC differentiation, biasing progenitors toward the osteoblast lineage at the expense of adipocyte formation. Intermittent PTH signaling (via cAMP/PKA pathways in stromal cells) promotes osteoblast differentiation and reduces marrow adiposity, contributing to PTH’s net anabolic impact on bone microarchitecture [54]. Moreover, PTH receptors have also been identified in blood vessels: PTH1R activation in vascular smooth muscle cells causes acute vasodilation and has been shown to reduce vascular oxidative stress and pro-calcific signaling [55,56,57]. These vasoprotective effects (including inhibition of Wnt/β-catenin-driven vascular calcification) imply that PTH/PTHrP signaling in arterial walls may counteract arteriosclerotic processes [58]. Collectively, while PTH’s primary role is in mineral metabolism, it also exerts influence on pancreatic insulin release, bone marrow stromal cell allocation, and vascular function—underscoring the hormone’s systemic scope of action.

### 2.4. The Transition to Secondary Hyperparathyroidism in Chronic Kidney Disease

CKD fundamentally disrupts the normal calcium–phosphate–PTH axis, precipitating SHPT. As glomerular filtration rate (GFR) declines, phosphate excretion is reduced, leading to phosphate retention; simultaneously, renal activation of vitamin D (calcitriol) falls, resulting in vitamin D deficiency and hypocalcemia. These changes remove the usual suppression on PTH: low calcium and high phosphate directly stimulate PTH secretion, while the lack of calcitriol implies diminished negative feedback on the parathyroids [28,59]. The bone-derived hormone FGF-23 rises early in CKD in response to phosphate accumulation and initially acts to inhibit PTH synthesis [28,29]. However, due to downregulation of the co-factor Klotho in uremia, the parathyroid glands become relatively resistant to FGF-23’s inhibitory signal [30]. Combined with progressive calcitriol deficiency, this allows PTH levels to climb unabated. The result is compensatory parathyroid hyperplasia and chronically elevated PTH secretion—the hallmark of SHPT.

Over time, sustained PTH elevation in CKD patients drives abnormally high bone turnover (remodeling rates), as the normal checks and balances on PTH (calcium, calcitriol, FGF-23) are impaired. Moreover, the uremic milieu and chronic PTH overstimulation can lead to a form of target-organ “resistance.” In advanced CKD, PTH’s biological effectiveness is blunted in part because a large fraction of circulating PTH is made up of inactive C-terminal fragments (due to reduced renal clearance) [33]. These fragments not only lack PTH1R agonism but may actively antagonize PTH action via the C-terminal PTH receptor on osteocytes, as discussed above [33,34]. Thus, despite very high circulating PTH levels, end-organs like bone sometimes respond less robustly than expected in uremia—a paradox attributable to receptor desensitization and the accumulation of inhibitory PTH fragments. Additionally, non-receptor mechanisms such as oxidative modification of PTH might further reduce its activity in CKD, although this is an area of ongoing research [60,61,62].

The impact of long-standing SHPT on the skeleton is profound. Chronic PTH excess produces the high-turnover form of renal osteodystrophy described as “osteitis fibrosa cystica”. Histologically, there is accelerated bone remodeling with excessive osteoclastic resorption and compensatory osteoblastic formation. This manifests as increased endosteal and intracortical bone resorption with resultant cortical thinning, alongside an expansion of unmineralized osteoid surfaces due to rapid but incomplete bone formation. The overall bone mass is reduced (particularly cortical bone, which constitutes the bulk of skeletal mass), and the bone that is present is of poorer quality (woven, undermineralized) [3,4].

**Figure 2:**
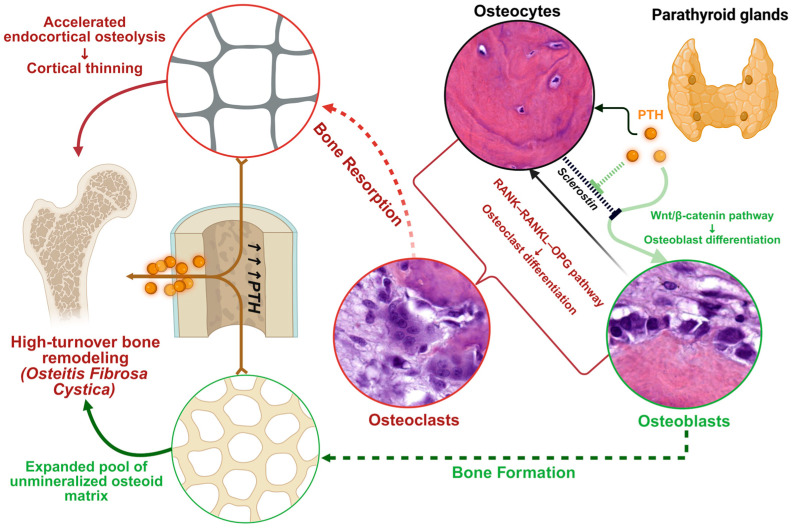
Physiological Parathyroid Hormone Bone Tissue Signaling and the Pathogenesis of High-turnover Bone Disease in Hyperparathyroidism. This schematic illustrates how PTH overstimulation drives high-turnover osteodystrophy. (1) PTH signaling cascades: PTH binding to PTH1R on osteoblasts/osteocytes activates cAMP/PKA and PKC pathways, enhancing pro-osteoblast Wnt/β-catenin signaling (via sclerostin suppression) and increasing osteoblast production of RANKL (while reducing OPG). (2) Osteoclast activation: RANKL from osteoblasts binds RANK on precursors, promoting osteoclast formation and bone resorption. Excess PTH thus causes robust osteoclastic endocortical resorption, contributing to cortical thinning and porosity. (3) Osteoblast response: Concurrently, PTH-stimulated osteoblasts deposit large amounts of new osteoid; however, in the chronic high-PTH state, this osteoid is only partially mineralized, leading to an expanded osteoid seam and woven (disorganized) bone formation. The balance of remodeling is shifted such that both bone resorption and formation are accelerated, but the newly formed bone is structurally inferior. Collectively, these processes underlie high-turnover bone disease in SHPT (osteitis fibrosa cystica), where skeletal integrity is compromised by both overt bone loss and impaired mineralization. Illustration created using BioRender [63].

Figure 2 illustrates these mechanisms and pathologic changes graphically. In the schematic, PTH’s actions on bone at the cellular level are depicted, including stimulation of osteoblasts and osteocytes to produce RANKL (which promotes osteoclastogenesis) and to reduce sclerostin (activating Wnt/β-catenin pathways), ultimately increasing both bone resorption and formation. Chronically elevated PTH is shown leading to accelerated endocortical osteolysis (bone breakdown at the inner cortical surface) and loss of cortical thickness, while simultaneously causing accumulation of osteoid (unmineralized matrix) on bone surfaces due to excessive, disorganized formation. These changes result in the structurally weak, high-turnover bone typical of advanced SHPT.

### 2.5. Pathogenesis of Hungry Bone Syndrome

HBS, the severe post-PTX hypocalcemia, arises directly from the preoperative SHPT skeletal state described above. The high-turnover bone with extensive unmineralized osteoid and expanded osteoblast populations creates a skeleton with massive capacity for mineral uptake. PTX abruptly removes the driving force for this high turnover. Immediately after the hyperactive parathyroid tissue is excised, circulating PTH levels plummet (often dropping by >70–90% within hours) and remain low [31]. The sudden withdrawal of PTH has two key effects on bone cell dynamics: it halts osteoclast-mediated resorption and therefore stops calcium release from bone, and it allows osteoblastic bone formation to proceed unopposed by any concurrent resorptive activity [31]. Osteoclasts, which have a short lifespan, become quiescent and undergo apoptosis in the absence of RANKL stimulation, whereas osteoblasts continue to function for a time, eagerly depositing mineral into the previously laid osteoid scaffold. The consequence is an acute and massive flux of calcium (and phosphate) out of the blood and into bone. Patients typically develop hypocalcemia within the first 2–3 days post-PTX as their serum calcium drops to a nadir (often <2.0 mmol/L or <8 mg/dL), frequently accompanied by hypophosphatemia and hypomagnesemia due to the skeletal uptake of minerals [21,24].

Clinically, this manifests in symptoms ranging from mild neuromuscular irritability (peri-oral tingling, muscle cramps) to severe complications such as tetany, seizures, laryngospasm, and cardiac arrhythmias [7,8]. In the most extreme cases, the profound hypocalcemia of HBS can be life-threatening, necessitating intravenous calcium infusions, cardiac monitoring, and intensive care support [7,8]. Notably, the bone formation drive remains elevated in the immediate postoperative period—BALP and other formation markers often continue to rise after PTX—indicating that osteoblasts are still active even as osteoclast activity has waned. This discrepancy underscores the molecular and cellular trajectory of HBS: the preoperative high-PTH milieu primes the skeleton with abundant formation surfaces and active bone-building cells, and when PTH is removed, the skeleton avidly mineralizes at the expense of circulating calcium. HBS thus represents the clinical culmination of uncoupled bone remodeling: ongoing formation despite abrupt cessation of resorption. Careful preoperative risk assessment and postoperative monitoring are therefore critical in these patients to anticipate and manage the profound calcium shifts that define HBS.

## 3. Perioperative Dynamics of Bone Turnover and Serum Markers

The temporal patterns of BTMs following PTX provide critical insights into both the pathophysiology of HBS and optimal monitoring windows. Understanding these dynamics enables clinicians to anticipate complications and tailor interventions based on expected biochemical trajectories rather than reacting to established hypocalcemia.

**Figure 3:**
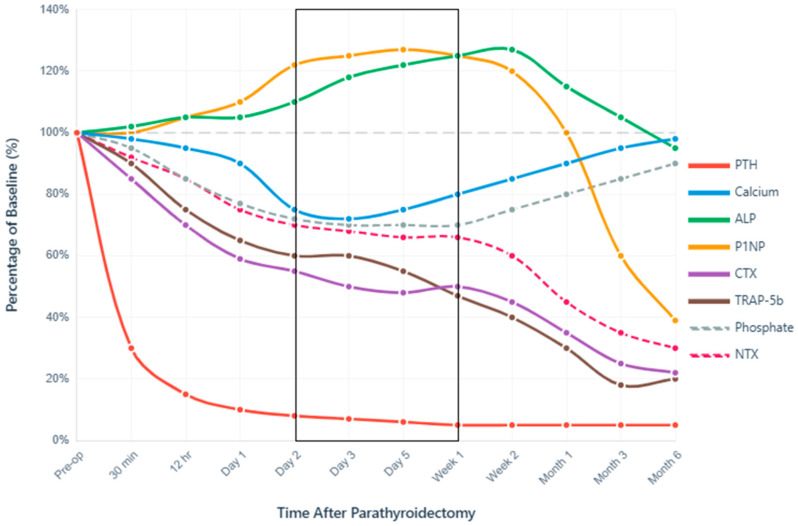
Perioperative Dynamics of Bone Turnover Markers After Parathyroidectomy. Normalized temporal changes in serum biomarkers expressed as percentage of baseline values (100% = preoperative level). The *x*-axis uses a logarithmic-like scale to capture both immediate intraoperative changes (minutes) and long-term recovery (months). PTH demonstrates immediate decline, confirming surgical success, while calcium nadir at 24–72 h defines the critical monitoring period, extended up to the first week post-PTX (black frame). The paradoxical early increase in bone formation markers (P1NP, ALP) despite PTH suppression, coupled with rapid resorption marker suppression (CTX, TRAP-5b), creates a favorable anabolic window for skeletal remineralization. Data synthesized from multiple prospective cohort studies and meta-analyses [21,23,24,71,73,74,75].

PTH demonstrates the most rapid response to successful gland removal among all markers. Due to intact (i)PTH’s short half-life of 2–5 min [31], real-time confirmation of surgical success can be ascertained intraoperatively, thus further guiding PTX surgical strategy [64]. In fact, intraoperative iPTH decline follows predictable kinetics, with the Miami criterion (>50% decline from peak pre-excision level at 10 min [65]) being achieved in a majority of cases, i.e., depending on baseline SPHT severity and surgical technique—subtotal versus total PTX +/− autograft. Thus, within 12 h post-PTX, mean relative PTH generally reaches a ~70–85.0% decline from baseline, with some cases showing an even more significant 98% reduction (from 383.7 to 6.5 pg/mL) [66]. Figure 3 illustrates the overall perspective: post-PTX PTH level drops to ~30% of baseline within 30 min and continues to decline to 10% or less by 24 h.

The first 72 h post-PTX represent the highest-risk window for symptomatic hypocalcemia. Serum calcium typically reaches its nadir between days 2–3 postoperatively, declining to ~72–75% of baseline values, specifically in those developing HBS, i.e., Ca^2+^ nadir < 8.4 mg/dL (<2.1 mmol/L), within the first 3 days post-PTX, lasting for ≥4 days and/or requiring IV Ca^2+^ for symptoms [21,24]. This timing proves remarkably consistent across populations, with 72.9% of patients achieving calcium normalization within 48 h and additional patients normalizing by 72 h [66]. The incidence of biochemical hypocalcemia varies dramatically between PHPT (42%) and SHPT (97%), though only 45–51% of those with biochemical changes develop symptoms [67]. Figure 3 illustrates this critical period (days 2–7) within a black frame, highlighting the moment when calcium reaches its lowest point, while bone formation markers paradoxically increase.

Phosphate dynamics differ between primary and SHPT, reflecting underlying pathophysiology. PHPT typically presents with hypophosphatemia (<2.5 mg/dL) due to PTH-induced phosphaturia [68], while SHPT patients often have hyperphosphatemia (>2.0 mmol/L) [69]. As seen in Figure 3, phosphate drops to 77% of baseline (from 2.28 to 1.75 mmol/L) on day 1 (*p* < 0.0001) post-PTX, with 38.7% developing more severe hypophosphatemia (<0.8 mmol/L) within the first month [24]. Thereafter, normalization timelines will vary considerably—most patients stabilize within 2 weeks, but severe cases require up to 52 weeks (mean 10.6 weeks in dialysis patients) [70]. This prolonged recovery reflects massive phosphate uptake by remineralizing bone, particularly in patients with preoperative bone disease severity.

A striking feature of post-PTX dynamics is the paradoxical increase in bone formation markers despite PTH suppression. As seen in Figure 3, ALP initially rises by 18–20% by day 3, usually peaking around 120–127% of baseline at 2 weeks, before gradually declining. By 3 months, ALP levels will normalize in 54.8% of cases, respectively, in 88.7% at 1 year post-PTX [24]. This counterintuitive pattern reflects continued osteoblast activity despite removal of the PTH stimulus, with the magnitude of the ALP surge correlating with total calcium supplementation requirements [24]. In HBS cases, an initial minor dip in serum levels precedes this ALP surge, which will be more aggressive and peak higher (potentially reaching 200% in severe cases), with higher overall values at different time-points (day 5, day 7 and 1 month [24]), reflecting intense mineralization of vast pre-deposited osteoid reserve pools—indicative of preexisting bone disease severity.

Similarly, P1NP shows an initial increase of 22% at day 2, and 27% at day 5 (see Figure 3), representing enhanced osteoblast activity that persists despite PTH withdrawal [71,72]. This initial rise contrasts sharply with the long-term trajectory, which shows a 61% decline from baseline at 6–12 months [73].

The uncoupling of bone formation and resorption creates the metabolic environment for HBS. While formation markers (ALP, P1NP) initially increase or remain elevated, resorption markers show immediate and sustained suppression. CTX decreases 21–41% within 24 h (*p* < 0.05) [71], with sustained suppression reaching 78% reduction at 6–12 months [73]. Conversely, TRAP-5b shows a more dramatic response with 52.89% decline by week 1 (significantly greater than NTX’s 34.42% reduction, *p* < 0.001) [71], ultimately achieving an 82% reduction at 3 months in surgically treated SHPT patients [74]. TRAP-5b’s independence from renal clearance makes it particularly valuable in CKD patients, maintaining interpretability across all stages of kidney disease.

This uncoupling creates an “anabolic window” lasting several months, during which bone formation significantly exceeds resorption. Histological studies confirm near-complete osteoclast disappearance from cancellous bone within one week, while cortical bone shows slower recovery [75]. The duration and magnitude of this anabolic phase correlate with preoperative disease severity and postoperative calcium requirements.

Recovery trajectories differ substantially between primary and SHPT. PHPT shows BMD improvements of 1–8% across all sites over 2 years, with lumbar spine responding most favorably [76]. SHPT patients experience more dramatic changes with BMD improvements of 7–23%, particularly in hemodialysis populations [77]. However, these patients face a higher HBS risk (20–70% versus 4–13%), requiring intensive postoperative monitoring [78]. Notably, 12–43% of patients maintain elevated PTH at 3 months despite normocalcemia, and 3–46% show persistent elevation beyond 6 months [79]. This eucalcemic PTH elevation correlates with preoperative levels > 225 pg/mL and vitamin D deficiency, suggesting ongoing skeletal recovery processes rather than surgical failure [80].

These temporal patterns provide actionable insights for perioperative management. The predictable calcium nadir at 48–72 h mandates intensive monitoring during this period, with prophylactic supplementation initiated based on early trajectory rather than waiting for severe hypocalcemia. The persistence of elevated bone formation markers despite PTH suppression explains why calcium requirements often escalate in the first week despite aggressive initial supplementation. Understanding that complete skeletal recovery requires months to years helps set appropriate expectations for both clinicians and patients regarding the duration of supplementation and monitoring.

## 4. Classification and Performance Metrics for Contemporary Predictors of Hungry Bone Syndrome

Multiple serum markers reflect bone metabolic activity. This section reviews the current major biomarkers—their biological roles, perioperative behavior, and predictive performance—investigated in the context of HBS. Table 1 provides an overview of key performance metrics (e.g., optimal cutoff values, sensitivity/specificity, predictive values) for selected key BTMs, as reported in representative studies. Notably, in advanced CKD, some markers require careful interpretation due to altered clearance. In general, markers not significantly affected by renal excretion (e.g., BALP, iP1NP, TRAP-5b) maintain better predictive accuracy [81,82] than those accumulating in renal failure (e.g., CTX, NTX) [83] and are thus preferred for assessing bone turnover in dialysis patients [84]. Indeed, expert consensus recommends BALP and TRAP-5b as reference markers for CKD-related bone disease, since PTH alone is not sufficient to determine turnover [4,85].

For fair comparison between BTM results, studies require standardized methods [96,97] and consistent HBS definitions. Only a few head-to-head assessments within the same HBS cohort have been reported thus far, yet these seemingly provide the most reliable insights. Notably, in dialysis patients with SHPT undergoing PTX, bone formation markers—especially ALP and BALP—have consistently outperformed other turnover markers in predicting HBS. Recurrently, it has been observed that although both formation and resorption markers (including NTX, CTX, and TRAP-5b) were markedly elevated in severe SHPT, only ALP emerged as an independent predictor of post-PTX HBS (alongside low calcium) on multivariate analysis [21]. Notably, some studies report that a high preoperative ALP is a better risk indicator for HBS than even PTH itself [24], underscoring the superior predictive value of traditional BTMs (particularly ALP/BALP) compared to other indices in this setting. However, it is important to note that the diagnostic criteria for HBS were not uniform across studies (e.g., varying calcium nadir thresholds, clinical manifestations and/or calcium IV supplementation requirements), which inherently hinders direct comparison between performance results from different sources, for both individual BTMs and combined risk models. Even so, beyond methodological inconsistencies, cost-effectiveness represents a concern, as the isolated incremental benefits of novel BTMs would rarely justify their costs in real-world scenarios.

Overall, current predictive tools and biomarker-based stratification schemes have significant clinical value in managing post-PTX hypocalcemia. High preoperative BTMs—notably PTH and ALP—consistently identify patients at greatest risk of HBS [86,87]. For example, SHPT patients with PTH levels exceeding ~1000 pg/mL and/or ALP > 150 U/L face a markedly elevated HBS risk [86]. However, the predictive power of any single parameter is only moderate; sensitivity and specificity plateau in the 70–80% range for isolated cut-offs [78,86], as seen in Table 1. This has motivated the development of combined risk models that integrate multiple predictors to improve discrimination. Indeed, multivariate tools can raise the area under the ROC curve (AUC) into the 0.85–0.90+ range, outperforming individual biomarkers [86]. In practical terms, leveraging both conventional metrics (e.g., extreme PTH/ALP elevations, hypocalcemia) and newer markers (e.g., P1NP, TRAP-5b) within an interpretive scoring system may further facilitate clinicians to pinpoint truly high-risk patients before they develop severe hypocalcemia [21,23,86,89]. Such biomarker-based risk stratification adds clear value: it enables targeted prophylactic calcium/vitamin D therapy, intensive monitoring, and triage of resources (e.g., ICU beds) to those most in need [7,8,9]. In summary, while no single laboratory value perfectly predicts HBS, the judicious combination of multiple preoperative indicators into validated risk models may greatly enhance our ability to forecast this syndrome and tailor preventive management.

## 5. Combined Biomarker Approaches and Risk Prediction Models

### 5.1. Statistical Methods and Model Development

Early HBS risk models were grounded in classical biostatistical techniques. Retrospective cohorts identified independent risk factors via multivariable logistic regression, yielding simplified scoring systems or nomograms [24,86,98]. Categorical cut-offs were often chosen for clinical simplicity (e.g., dichotomizing “high” vs. “normal” PTH levels) at the expense of some granularity. Recognizing this trade-off, some models assign weighted point values or use continuous predictors to maximize predictive resolution [24,87,98]. Across studies, internal validation is typically performed with resampling techniques (bootstrap or k-fold cross-validation) to guard against over-fitting [24,86,87]. Calibration analysis (comparing predicted vs. observed HBS frequencies) is another key step to ensure the model’s risk estimates are reliable when applied clinically. Where possible, external validation on independent patient cohorts is pursued to test generalizability, though such data remain limited for most HBS tools.

Recently, more complex approaches—including ML algorithms—have been explored to incorporate a high-dimensional array of clinical and biochemical features [99]. While these advanced models (e.g., ensemble tree methods) can potentially boost accuracy, they sacrifice interpretability and have thus far been constrained by relatively small sample sizes [99]. In practice, clinicians favor parsimonious risk scores that are easy to calculate at the bedside. Ultimately, whether simple or complex, a useful HBS prediction model must demonstrate robust discrimination (high sensitivity/specificity or C-statistic), good calibration, and clinical utility (e.g., high positive or negative predictive value—PPV or NPV- for guiding management). Table 2 below summarizes the leading multivariate risk models for HBS, highlighting their components, performance, and validation status.

To sum up, the development and validation of prediction models for HBS has evolved considerably, with approaches ranging from simple two-variable scoring systems to complex ML algorithms. Understanding the methodological strengths and limitations of these models is essential for appropriate clinical application.

### 5.2. Key Multivariate Risk Prediction Models for Hungry Bone Syndrome

#### 5.2.1. NYU Langone 2-Point Preoperative Scoring System

This simple scoring tool was developed on a small single-center cohort of dialysis patients with SHPT (*n* = 33) undergoing PTX [86]. Investigators applied logistic regression with a Boruta feature selection algorithm, identifying two preoperative biomarkers—ALP and iPTH—as key HBS predictors [2]. Each variable is dichotomized using a high-risk cutoff (ALP > 150 U/L = 1 point; PTH > 1000 pg/mL = 1 point), yielding a total score from 0 to 2, i.e., stratifying patients as low (0), high (1), or very high risk (2) for HBS [86].

Despite its simplicity, the NYU 2-point score showed excellent predictive performance in its initial validation. It achieved 96.8% overall accuracy with 100% sensitivity and 94.1% specificity for HBS [86]. Notably, a 0-point result had a 100% NPV, i.e., HBS did not occur if both PTH and ALP were below thresholds, whereas a 2-point score predicted HBS with ~100% PPV [86]. As seen in Table 2, even a single risk factor (score = 1) was associated with markedly elevated risk (~94% PPV), warranting closer monitoring.

Clinically, the NYU score functions as an immediate bedside tool, requiring only two routine labs. Its binary cut-points allow instant stratification during preoperative assessment. The trade-off for this ease-of-use is some loss of granularity—it does not distinguish how high a patient’s PTH or ALP is once beyond the cutoff. Regardless, the score’s high NPV allows confident routine post-PTX management when both markers are low, whereas a full 2-point grade reliably flags patients for intensive calcium monitoring and supplementation. However, the model’s derivation from a small cohort and only informal external validation to date, limit certainty of its generalizability. Even so, its impressive performance and simplicity make it a promising screening tool for the perioperative management of HBS.

#### 5.2.2. Gao Nomogram

In China, Gao et al. developed a nomogram-based risk model using data from 75 dialysis patients with SHPT [8], using multivariate logistic regression to integrate two continuous predictors: preoperative serum iPTH and ALP [87]. Both inputs are treated as continuous variables, avoiding artificial cutoffs and thereby preserving nuanced risk gradation. The resulting model provides a patient-specific risk probability (0–100%) of post-PTX HBS, calculated via a simple logistic equation (see Table 2), which can be applied without special software.

The Gao nomogram demonstrated excellent discriminative ability, with an HBS incidence of 42.7% among the study population. In the derivation cohort, the model’s C-index was 0.943 (95% CI 0.892–0.994)—one of the highest reported for HBS prediction. Internal validation with 1000-bootstrap resampling confirmed robust performance and good calibration (Hosmer–Lemeshow χ^2^ = 3.405, *p* = 0.474) [87]. Of note, PTH and ALP each had strong univariate predictive power in this dataset (AUC 0.87 for PTH, 0.93 for ALP)—and their combination yielded only a modest incremental gain (~1–2% increase in AUC) over the better single marker. This suggests that while the two-marker model was extremely accurate, a large share of its predictive strength came from ALP alone. No external validation has been reported yet for this nomogram.

One practical difference from the NYU score is that Gao’s model derived mathematically optimal cut-points that were higher (PTH ~2433 pg/mL, ALP ~289 U/L by Youden’s index [87]) than the NYU’s round-number thresholds [86], as seen in Table 1 and Table 2. In effect, the nomogram maximizes statistical sensitivity/specificity, whereas the NYU score prioritizes ease-of-use and generalizability in exchange for a slight trade-off in precision [86,87]. For clinicians, the Gao model’s high accuracy is appealing, but its real-world utility will depend on validation in broader populations and ensuring that busy providers can conveniently perform the risk calculation. Given its simplicity (only two variables) and strong performance, this nomogram can serve as a valuable intermediate step—more individualized than a fixed cut-off score, yet far simpler than multi-factor ML models.

#### 5.2.3. Wang Nomogram

Another Chinese nomogram, developed by Wang et al. at West China Hospital, on a cohort of 114 dialysis patients with severe SHPT, who underwent total PTX and showed a 76.3% incidence of HBS, proposed a more complex layout. This logistic regression-based perioperative tool incorporates the continuous values of four distinct factors: (1) iPTH; (2) BALP; (3) corrected serum calcium; and (4) total weight of excised parathyroid glands (in grams) [24]. Each of these contributes to a point score on a printed nomogram; the total points map to an estimated probability of HBS ranging from 0 to 100% [24]. Uniquely, it includes gland weight as an anatomic measure of disease burden (i.e., gland hyperplasia), though this parameter is only available intraoperatively, making the model partly postoperative in application (though all other inputs are preoperative).

Biomarker synergy occurs when predictors capture orthogonal information. PTH reflects disease severity and gland hyperfunction, while ALP indicates bone turnover intensity and osteoblast activity. Their combination provides complementary perspectives on parathyroid-bone axis dysfunction. Similarly, preoperative calcium (inverse predictor) and phosphate (direct predictor) reflect different aspects of mineral homeostasis [100].

This four-factor nomogram showed high discriminatory power (AUC~0.92) outperforming individual predictors: iPTH (~0.84), BALP (~0.78), gland weight (~0.70), and calcium (~0.65). Herein, each factor added some predictive value, with PTH and BALP contributing the most. Internal validation via bootstrap resampling (1000 iterations) showed good calibration [24]. However, to date, no large external validation has confirmed its performance in other populations. Conversely, the nomogram’s heightened complexity did not produce a dramatically higher AUC than simpler two-marker models, suggesting possible diminishing returns beyond the top 2–3 predictors.

Cross-cultural validation remains particularly sparse. Indeed, multiple high-performing models originate from Asian centers (China, Hong Kong, Thailand), where reported HBS incidence ranges from 42.7% to 87.8% [24,87,101]. Western populations show lower incidence (19.4% in USRDS data), possibly reflecting differences in patient selection, surgical timing, dialysis practices, or genetic factors [98]. The He et al. model achieved C-index of 0.866–668 0.867 using separate training and validation cohorts, representing one of few studies with formal internal-external validation [102]. Conversely, online dynamic nomograms, as developed by Cao et al., further enhance accessibility while maintaining the transparency of logistic regression coefficients [103].

All in all, the Wang nomogram represents a more detailed risk stratification tool that sits between simple scores and “black-box” ML. It leverages both biochemical markers and surgical pathology information to refine risk estimates. In practice, using the model requires BALP measurement (not routine in all laboratories) and intraoperative gland weight data. However, relying on more variables increases complexity without a proportionate leap in accuracy for most patients. Therefore, clinicians might find the Wang model most useful in borderline cases or in research settings, while recognizing that much of HBS risk can often be gauged from a few key labs alone.

#### 5.2.4. USRDS Risk Score

This HBS risk score was developed using the large-scale United States Renal Data System (USRDS) registry, analyzing 17,074 PTX cases from within a national dialysis population [98]. The model purposefully uses only readily available clinical variables, without any biochemical data, making it applicable even when lab values are unavailable. Five preoperative factors were assigned point values based on their statistical weight in predicting HBS (see Table 2):

- Age < 48 years—2 points;
- Dialysis duration ≥ 5 years—1 point;
- Evidence of renal osteodystrophy—1 point;
- Kidney transplant vintage ≥ 3 years—1 point;
- Elixhauser comorbidity score ≥ 5—1 point.

Higher scores correspond to increasing HBS probability: 0 points predicted ~8% risk, while 6 points predicted ~44% risk. Intermediate scores ranged from 1 to 2 points (~12–18% risk) to 3–4 points (~26–35% risk). The dataset was split 75%/25% into training and validation sets for internal performance testing. Excluding biochemical predictors, the USRDS score showed limited discrimination, with only ~46% accuracy, moderate sensitivity (75.6%), and low specificity (40.7%). The positive predictive value was only ~20%, generating many false-positives [98]. However, its strength lies in ruling out HBS: patients with 0 points almost never developed the syndrome, yielding a high NPV of 89.3%. The absence of key biomarkers (PTH, ALP) from the registry analyzed likely contributed to the model’s weaker predictive power.

To conclude, the USRDS risk score provides quick, bedside-applicable stratification requiring only patient history and clinical information, without any laboratory data. A score of 0 reliably indicates minimal HBS risk, potentially supporting decisions for shorter hospitalization or less intensive monitoring. Conversely, high scores have poor specificity and should not trigger aggressive intervention without corroborating biochemical evidence. Notably, patients who developed HBS had significantly higher ICU admission rates postoperatively (33.5% vs. 24.6%, *p* < 0.001) [98], highlighting the clinical significance of risk identification. In summary, the USRDS risk score is most useful for rule-out purposes and broad stratification when lab-based models are unavailable; it is an entry-level risk estimator that should ideally be combined with more specific measures for confirming truly high-risk cases.

#### 5.2.5. XGBoost Machine-Learning Model

Recently, ML approaches have been applied to HBS prediction to capture complex nonlinear patterns. Chai et al. evaluated several ML algorithms in a cohort of 181 dialysis patients with SHPT, finding that Extreme Gradient Boosting (XGBoost) performed best [99]. Their final model used five key features, selected from an initial candidate pool of 46 variables, via logistic regression and Boruta algorithm, namely: preoperative PTH, the intraoperative %PTH decline (from pre-incision to post-excision, reflecting the magnitude of immediate PTH drop), serum ALP, corrected calcium, and patient age [99]. The XGBoost model processes these continuous variables to output an individualized HBS probability, triaged into three risk categories for clinical interpretation—low risk < 20%, moderate 20–35%, and high risk ≥ 35% (see Table 2). Importantly, the model’s structure is nonlinear and non-parametric, meaning it automatically captures interactions or threshold effects that a simple logistic score would surely miss. The final tool was made available as a web-based application to facilitate user-friendly access in clinical settings.

In the validation cohort (30% of data), the XGBoost model achieved an AUC of 0.878 (95% CI 0.779–0.973), indicating strong discrimination. It balanced sensitivity and specificity well, as reflected by the high F1 score of 0.87 [99]. At the chosen risk threshold, the model correctly identified ~87% of patients who developed HBS (sensitivity 87.1%) while correctly recognizing ~82% of those who did not (specificity 81.8%). This performance exceeded that of traditional logistic regression in the same dataset—logistic models had lower AUCs (~0.68–0.83) and missed more HBS cases [99]. The XGBoost algorithm thus demonstrated an ability to extract slightly more predictive signal from the data, likely by accounting for interactions that linear models might not fully capture. It should be noted that these results are from a single-center study; no external validation, beyond the internal train-test split, has been reported yet. The model’s calibration was acceptable in the internal analysis, although careful monitoring of calibration in new populations will be needed, as ML models can sometimes show calibration drift when applied to different settings, i.e., parathyroid carcinoma XGBoost application with overprediction tendencies, despite acceptable calibration [104].

Overall, the XGBoost model represents the most complex risk prediction approach, with advantages including the ability to incorporate multiple variables and uncover non-obvious risk patterns, potentially improving sensitivity in identifying high-risk patients. The use of SHapley Additive exPlanations (SHAP) in this model provides clinicians with insights into individual predictions, displaying how much each factor contributed to the patient’s final risk score, thereby mitigating the “black box” issue and increasing clinician confidence in the ML model’s recommendations. On the downside, implementing the XGBoost score at the point of care is less straightforward. It requires either integration into electronic systems or use of an online calculator, since one cannot manually compute the risk from a simple equation. Additionally, one of its inputs—the intraoperative PTH drop—is only available during surgery, which means preoperative risk planning cannot solely rely on this model (though the model could still be run intraoperatively to guide immediate postoperative management). In resource-limited settings or smaller centers, the parsability and immediacy of simpler scores (like NYU or Gao) may outweigh the modest accuracy gains of an ML approach. In summary, the XGBoost HBS model is a powerful tool that can augment risk assessment, particularly in specialized centers where the necessary data and computational support are available. It stands as a proof-of-concept that advanced analytics can achieve high accuracy (AUC ~0.88) within a limited feature set [99], even though its clinical role will most likely remain complementary to simpler bedside risk scores, providing additional risk insight, rather than replacing them. Further multi-center validation will determine how broadly applicable this approach is and whether its superior discrimination translates into better clinical outcomes for patients at risk of HBS.

## 6. Clinical Implementation, Guidelines, and Risk-Stratified Management Protocols

Translating HBS risk prediction into improved patient outcomes requires effective clinical implementation. In this final chapter, we explore how international guidelines and institutional protocols address (or overlook) HBS risk, and we discuss practical strategies for risk-stratified management.

### 6.1. Clinical Practice Guidelines for Post-Parathyroidectomy Management

The Kidney Disease: Improving Global Outcomes (KDIGO) 2017 Clinical Practice Guideline Update provides foundational recommendations for PTX in CKD. For patients with CKD G3a-G5D with severe HPT failing medical therapy, PTX is suggested (Grade 2B evidence) [4]. Post-operative monitoring recommendations specify serum calcium and phosphate measurements every 1–3 months in CKD G5/G5D patients, with PTH monitoring every 3–6 months [4]. During active treatment for CKD-MBD or after PTX, measurement frequency should increase to monitor for efficacy and adverse effects. The guidelines recommend avoiding hypercalcemia (Grade 2C) and correcting vitamin D deficiency using strategies recommended for the general population (Grade 2C) [4].

The 2016 American Association of Endocrine Surgeons (AAES) guidelines emphasize comprehensive preoperative evaluation, including 25-hydroxyvitamin D (25-(OH)D) measurements and supplementation for deficiency [105]. Calcium supplementation may be indicated postoperatively based on serum calcium levels and symptoms. All symptomatic patients warrant PTX, with postoperative evaluation for hypocalcemia and related symptoms. Follow-up assessment defining cure as eucalcemia beyond 6 months is recommended [105].

The 2018 KDOQI US Commentary on the KDIGO guidelines notes that PTH levels, corresponding to the KDIGO target of 2–9× the upper normal limit, translate to approximately 120–660 pg/mL, depending on the assay [106]. Mortality risk associations vary, with PTH levels of 400–600 pg/mL representing inflection points for increased all-cause mortality. PTX by expert surgeons effectively decreases PTH, calcium, and phosphorus in patients with acceptable surgical risk when medical therapy fails [106].

### 6.2. Risk-Stratified Management Protocols

#### 6.2.1. Prophylactic Calcium Supplementation Strategies

Risk-stratified supplementation protocols vary from universal prophylaxis to selective approaches based on prediction models. The NHS Scotland protocol initiates prophylactic treatment 3 days preoperatively for total PTX patients: calcium carbonate 25 mmol three times daily given outside mealtimes, plus alfacalcidol 2 micrograms daily, continuing postoperatively with dose adjustment based on twice-daily calcium monitoring [107].

PTH-guided protocols stratify patients by 4 h postoperative PTH levels: low risk (PTH > 30 pg/mL) receives supplementation only if symptomatic; medium risk (PTH 15–30 pg/mL) receives selective supplementation; high risk (PTH < 15 pg/mL) receives aggressive supplementation [108]. This approach significantly reduces postoperative hypocalcemia and hospital readmissions. Emerging ALP-guided protocols have shown promising results as well [109].

The reactive approach employed at Hong Kong centers begins with standardized IV calcium infusion (2720 mg elemental calcium daily, as 50 mL 10% calcium chloride in 450 mL normal saline) with calcium monitoring every 6 h [24]. If calcium drops to ≤2.1 mmol/L, an additional 272 mg elemental calcium bolus is administered. When calcium exceeds 2.4 mmol/L, IV infusion is temporarily held for 6 h, then resumed. Transition to oral calcium plus alfacalcidol or calcitriol occurs as soon as the patient is able to take oral meds, with IV therapy being gradually weaned, followed by discharge once serum calcium stabilizes [24].

#### 6.2.2. Monitoring Frequency Based on Risk Level

Standard post-PTX monitoring for SHPT includes intensive early surveillance. The first 48 h require calcium measurement every 6 h, with particular attention to the expected trough at approximately 2 weeks postoperatively when ALP peaks [24,109]. Days 3–14 typically require daily or every-other-day calcium, phosphate, and ALP measurements. Weeks 2–4 involve monitoring 2–3 times weekly, transitioning to weekly biweekly during months 1–3, then monthly through month 6 [24]

High-risk patients identified by prediction models (ALP > 300 U/L, PTH > 2400 pg/mL, NYU score of 2) warrant enhanced surveillance with consideration for central venous access [110]. ALP trends serve as a biomarker for bone formation intensity, as rising ALP strongly correlates with declining calcium (r = 0.76) [24,109].

#### 6.2.3. ICU Admission Criteria

ICU admission for post-PTX management remains more common in secondary versus primary HPT. Contemporary practice bases ICU admission on predicted HBS risk and early postoperative calcium trends. Specific indications include symptomatic hypocalcemia (tetany, laryngospasm, seizures), cardiac manifestations (arrhythmias, QTc prolongation), laboratory criteria (ionized calcium < 0.9 mmol/L, refractory hypocalcemia), and high-risk patient factors (ALP > 500 U/L, expected calcium requirements >10 g in first week, concurrent cardiac disease) [111,112,113].

USRDS data documented that 33.5% of HBS patients required ICU admission compared to 24.6% without HBS (*p* < 0.001), representing a 36% relative increase [98]. Patients with ALP < 150 U/L (0% HBS incidence) may receive routine ward care, while those with ALP > 300 U/L (~100% HBS incidence [87,88]) represent appropriate candidates for prophylactic ICU admission.

#### 6.2.4. Vitamin D Supplementation Protocols

Native vitamin D (cholecalciferol or ergocalciferol) supplementation addresses deficiency preoperatively, with ergocalciferol 50,000 units weekly commonly prescribed when 25-(OH)D levels fall below 32 ng/mL [114]. Postoperative maintenance typically involves 400–800 IU daily [114].

Active vitamin D analogs play a critical role in HBS prevention and management. Alfacalcidol protocols begin 2 micrograms daily starting 3 days before surgery, continuing postoperatively with dose adjustment based on calcium levels [24]. Calcitriol dosing for HBS prophylaxis typically starts at 2–4 micrograms daily during the perioperative period [24,111,114]. Treatment of established HBS may begin with 2–4 micrograms daily in divided doses, with severe HBS potentially requiring 4 micrograms daily for several days before down-titration [24,115]. Long-term chronic management usually starts at 0.25 mcg daily with up-titration every 4–8 weeks to 0.5–2 mcg daily [24,111,113,114,115,116].

### 6.3. Implementation Strategies in Clinical Practice

Successful HBS management programs employ standardized, risk-stratified preprinted order sets with automatic laboratory ordering protocols and alert systems for critical values [117]. Multidisciplinary approaches involve endocrinologists, nephrologists, specialized nursing, pharmacists, and nutritionists [117].

Electronic medical record (EMR) integration enables automated HBS risk score calculation from laboratory values, clinical decision support alerts, calcium trend monitoring dashboards, and automatic ordering protocols [117]. The Chai et al. web application for XGBoost predictions and the Cao et al. online dynamic nomogram exemplify digital tools enabling real-time risk estimation [99,103].

Protocolized care demonstrates substantial benefits. PTH-guided supplementation protocols reduced hypocalcemia rates from 16% to 2.3% and 30-day readmissions from 2.7% to 0.01%, while reducing hospital stays by 2–5 days in high-risk patients [108,117]. The Singapore General Hospital’s ALP-based prophylactic protocol successfully reduced severe hypocalcemia in 167 ESKD patients [109].

## 7. Conclusions

HBS remains a significant clinical challenge post-PTX in SHPT, with incidence rates ranging widely among current reports (20–70%). This variability underscores the importance of diagnostic criteria standardization for accurate preoperative risk stratification. Our review reports that both individual BTMs and multivariate HBS risk prediction models can effectively identify high-risk cases, requiring intensive perioperative management.

Among individual biomarkers, preoperative ALP and iPTH have been consistently reported as the strongest predictors of HBS, with ALP > 150–300 U/L and/or PTH > 1000–2400 pg/mL associating substantially elevated risk. Novel markers such as BALP and TRAP-5b offer incremental predictive value, especially in CKD, where traditional markers may be confounded by reduced renal clearance. Low preoperative calcium, younger age, prolonged dialysis vintage, and absence of parathyroid autotransplantation further refine risk assessment. Combined risk models—ranging from the simple two-variable NYU score to sophisticated ML algorithms (XGBoost)—show superior discrimination (AUC 0.87–0.95) compared to single markers, enabling more precise risk stratification.

However, these emerging HBS prediction tools still face persistent limitations. Most models derive from single-center studies with internal validation only, whereas cross-cultural external validation remains sparse, despite evidence of population-specific HBS incidence differences. Heterogeneity in HBS definitions across studies complicates comparisons between biomarker thresholds and/or model performance. The optimal balance between model complexity and clinical usability remains uncertain.

Translating risk prediction into improved outcomes requires standardized, protocol-driven care pathways. High-risk patients identified preoperatively should receive prophylactic calcium and active vitamin D supplementation, intensive postoperative monitoring (especially during the critical 48–72 h window when calcium reaches its nadir), and consideration for ICU-level care when severe HBS is anticipated. Conversely, validated low-risk stratification enables confident outpatient management or shortened hospitalization, optimizing resource allocation. However, it should be noted that, thus far, much of the available evidence (including several risk models) comes from single-center studies with limited sample sizes. Therefore, their performance requires validation in larger multi-center cohorts before widespread clinical adoption.

Future research priorities include: (1) prospective external validation of existing prediction models across diverse populations; (2) standardization of HBS diagnostic criteria; (3) cost-effectiveness analyses comparing protocol-driven prophylaxis versus reactive management; (4) investigation of emerging biomarkers such as FGF-23 and sclerostin; and (5) development of real-time clinical decision support tools integrated into EMR systems. By integrating biomarker-driven predictive strategies with evidence-based perioperative protocols, clinicians can substantially reduce HBS-related morbidity and improve outcomes for this high-risk population.

## Figures and Tables

**Table 1:** Established and Novel Bone Turnover Biomarkers as Predictors of Hungry Bone Syndrome—Summary of Evidence.

	Type	Name	Predictive Threshold	Performance Metrics	Specific Considerations	Limitations
Traditional Biomarkers	Regulatory	intact (i)PTH	>1000 pg/mL	Sensitivity: 100%; Specificity: 94.1%; Accuracy: 96.8%; PPV: 93.8–100%; NPV: 100% [86].	Part of 2-point scoring system (NYU). Mean PTH (2167.2 pg/mL) ↑ in HBS. Validated in CKD-associated SHPT (*n* = 33).	Pre-PTX calcimimetic therapy may ↓ PTH levels. Inter-assay variability (2–3× for the same sample). Possible “uremic PTH” resistance (↓ bone tissue responsiveness).
			>2433.1 pg/mL	AUC: 0.873; (95% CI: 0.785–0.961) [87].	Extremely ↑ threshold in Chinese dialysis patients (*n* = 75). Higher threshold needed for Asians.	
	Formation	Total ALP	>150 U/L	+PTH > 1000 pg/mL: Sensitivity: 100% Specificity: 94.1%; Accuracy: 96.8% [86].	↓ threshold for screening. Part of validated NYU 2-point score. Simple bedside calculation: ALP > 150 + PTH > 1000 = high PPV (~94%) for HBS.	Non-specific: Includes hepatic sources. Population variations: Different optimal thresholds dependent on ethnicity and disease severity. Requires additional validation in more diverse demographics. Very ↑ thresholds (~250–300 U/L) generally improve specificity, albeit with the price of lowering sensitivity = missing milder cases.
			>199.5 U/L	Sensitivity: 80.85%; Specificity: 82.61%; AUC: 0.871 [78].	Independent predictor in multivariate analysis (threshold from pooled data). Supported by a meta-analysis, yet inter-study heterogeneity exists.	
			>289.5 U/L	AUC: 0.926; (95% CI: 0.871–0.980) [87].	Highest single marker performance reported. Validated in a dialysis SHPT cohort (*n* = 75).	
			>340 U/L	Sensitivity: 100%; Specificity: 95%; PPV: 100% [88].	Protective if <340 U/L in PHPT (*n* = 29). Threshold may be too high for SHPT (most CKD patients never reach such levels pre-op).	
			↑ Pre-PTX mean	Δ HBS vs. non-HBS: 415 vs. 221 U/L (OR ≈ 1.005 per unit ↑; *p* = 0.008) [24,89].	ALP difference is clinically significant (≈2 × ↑ in HBS). Independent predictor in SHPT (*n* = 62). Wide range between groups: ↑ values strongly indicate HBS, but moderate ones may need context (bone vs. liver origin).	
	Minerals	Ca^2+^ (corrected)	<2.44 mmol/L * (≈9.5 mg/dL)	↓ Mean pre-PTX in HBS vs. non-HBS: 2.44 vs. 2.60 mmol/L (*p* = 0.001) [24].	↓ Pre-op Ca^2+^ = ↑ HBS risk, i.e., depleted skeletal stores and active uptake favor post-op “Ca^2+^ crash”. Consistently predictive for HBS (*n* = 62); part of risk models. Early post-op Ca^2+^ decline velocity = a rapid drop further flags onset.	Influenced by dialysis prescription, nutritional status, and vitamin D therapy. Diagnostic criterion post-PTX—consequence rather than predictor –, yet here the magnitude of the drop is what counts. Best interpreted alongside PTH and ALP for HBS risk assessment.
			Δ ≈ −0.96 mg/dL	Pre-PTX mean Ca^2+^ HBS vs. non-HBS (*p* = 0.004) [86].	↑ Pre-PTX Ca^2+^ is protective. HBS cases start ~1.0 mg/dL lower in baseline Ca^2+^ on average, e.g., 9.6 vs. 10.4 mg/dL (*n* = 36, *p* = 0.01). In dialysis cases (*n* = 33), ↓ Ca^2+^ was one of the only significant pre-PTX predictors for HBS. Moderate stand-alone PV: HBS with normal baseline Ca^2+^, especially in adynamic bone disease.	
		PO_4_^3−^	Δ ≈ +3.5 mg/dL	Pre-PTX mean PO_4_^3−^: HBS vs. non-HBS (*p* < 0.001) [86].	Noted primarily in non-CKD settings—e.g., in PHPT, HBS patients had ↑ PO_4_^3−^. In SHPT, PO_4_^3−^ reflects severity, but has not shown strong HBS PV. Pre-PTX ↑ PO_4_^3−^ may predict HBS risk. In dialysis patients, despite HBS cases having ↑ mean PO_4_^3−^, when accounting for PTH and ALP, PO_4_^3−^ was not an independent HBS predictor.	Dialysis-dependent changes. PO_4_^3−^ binders affect levels. Most dialysis patients present with ↑ PO_4_^3−^, limiting discriminatory power in CKD-SHPT. Not reliable as a sole predictor—inconsistent HBS PV.
Novel	Formation	BALP	>42 μg/L *	Sensitivity: 79%; Specificity: 82%; AUC: 0.80–0.85 [23].	More specific than total ALP. Less biological variability than PTH. Independent predictor in Wang nomogram (*n* = 114). Peaks ~2 weeks post-PTX (after Ca^2+^ nadir), indicating continued osteoblastic activity. Useful when ALP accumulation is a concern (liver disease or dialysis ineligibility).	↑ costs/↓ availability of assays have prevented widespread clinical adoption.
		P1NP (intact)	>80 μg/L *	No HBS PV in CKD-SHPT reported. Only one PHPT study: *p* = 0.427 [90].	Minimal circadian variation. Normal range: 15–80 μg/L. A sensitive formation marker in osteoporosis. Promising direction, despite not correlating with HBS risk thus far, in CKD-SHPT.	Not validated for HBS. KDIGO: not recommended in CKD. Accurately reflects osteogenesis activity.
		Osteocalcin	NE	Mixed results: ↓ Pre-PTX in HBS; OR: 1.001 per ng/mL for ↑ hospital stay; ↑ Post-PTX: 264 → 478 ng/mL (*p* < 0.001) [89].	↑ Osteocalcin = ↑ bone turnover. Independent pre-PTX predictor for ↑HBS hospitalization, alongside ALP, in large dialysis cohort (*n* = 260). Dual marker (formation + resorption). Significant surge post-PTX, reflecting osteoblast activity boom.	Significant circadian variation (morning fasting sample). Vitamin K dependent. Accumulates in ESRD.
	Resorption	CTX/NTX	NE	No HBS PV in CKD-SHPT reported. HBS vs. non-HBS: CTX (*p* = 0.110); NTX (*p* = 0.273) [21].	Normal range: CTX 0–0.3 μg/L; NTX 15.1–36.4 μg/L. Pre-PTX CTX: 6.0 (4.86, 6.0) μg/L; Pre-PTX NTX: 1200.0 (1057.75, 1232.2) μg/L Post-PTX: CTX ↓ significantly to 1.56 μg/L (*p* < 0.001); NTX did not (*p* = 0.794)—possibly due to hemodialysis. ↑ threshold suggests severe turnover. ~78% drop at 6–12 months post-PTX, mirroring the decline in bone resorption activity.	Limited utility in CKD—renal clearance: ↑ CTX/NTX with ↓ GFR. ↑ biological variability + ↑ circadian variations (morning fasting sample). Results differ by HPT subtype. NTX affected by hemodialysis (56.6% ↓/session).
		TRAP-5b	>10–15 U/L *	↑ Pre-PTX in HBS vs. non-HBS: 12.44 vs. 6.24 IU/L (*p* = 0.001, *n* = 115); Marked post-PTX ↓: 7.20 ± 4.11 IU/L (*p* < 0.001) [21].	Normal range: 3.25 ± 0.59 U/L. Pre-PTX mean: 11.89 ± 6.30 IU/L. Specific to osteoclastic activity. Post-PTX, prompt drop (~40% in 3 days) as bone resorption abates. Correlated with pre-PTX iPTH (r = 0.783, *p* < 0.001) and IV Ca^2+^ supplementation dose (r = 0.445, *p* < 0.001). Despite univariate significance, not an independent HBS predictor when accounting for ALP and Ca^2+^.	Not affected by renal clearance (metabolized by liver)—maintains reliability in ESRD. Still considered only a research marker for CKD patients. No validated HBS cutoff to date (insufficient data). More studies needed to define predictive thresholds.
	Regulatory	FGF-23	NE	Research use only. No HBS-specific data.	Markedly ↑ in CKD (100–1000× ULN) [91], due to PO_4_^3−^ retention [92]. May ↓ post-PTX in SHPT, as PO_4_^3−^ normalizes. A case of tertiary HPT in X-linked hypophosphatemia noted severe HBS despite inherently ↑ FGF-23 levels [93].	Very costly, specialized assay. Not widely accessible. Unclear utility: Δ among HPT subtypes. So far, not used for HBS risk stratification.
		Sclerostin	NE	Discriminates turnover. No HBS-specific metrics.	↑ in CKD (3–5× ULN in dialysis patients) [39]. Suppressed by chronically ↑ PTH—sclerostin levels lower than they would be; hence levels tend to ↑ after PTX removes suppression (in CKD-MBD) [94]. May correlate with bone pain (limited CKD evidence).	Up to 4× assay variability between platforms [7,95]. Research marker currently—not used clinically for HBS prediction.

*—Theoretical, proposed from reference ranges, not validated; ↑ = Increased/elevated; ↓ = Decreased/reduced; Δ = Difference/change between groups; *n* = number of cases; NE = Not Established; iPTH = intact parathyroid hormone; ALP = alkaline phosphatase; BALP = bone-specific alkaline phosphatase; P1NP = procollagen type I N-terminal propeptide; Ca^2+^ = corrected calcium; PO_4_^3−^ = phosphate; CTX = C-terminal telopeptide; NTX = N-terminal telopeptide; TRAP-5b = tartrate-resistant acid phosphatase 5b; FGF-23 = fibroblast growth factor-23; HBS = hungry bone syndrome; PTX = parathyroidectomy; SHPT = secondary hyperparathyroidism; PHPT = primary hyperparathyroidism; CKD = chronic kidney disease; ESRD = end-stage renal disease; KDIGO = Kidney Disease: Improving Global Outcomes; AUC = Area Under Curve; OR = Odds Ratio; PPV = Positive Predictive Value; NPV = Negative Predictive Value; CI = Confidence Interval; PV = Predictive Value; NYU = New York University; ULN = upper limit of normal.

**Table 2:** Comparison of selected HBS risk prediction models, their components, and performance.

	Model Components	Scoring/Calculation	Risk Categories	Performance	Validation
NYU 2-Pt. Score [86]	ALP > 150 U/L (1 pt.); PTH > 1000 pg/mL (1 pt.).	Total score: 0–2 points.	0: Low risk; 1: High risk; 2: Very high risk.	Accuracy: 96.8%; Sensitivity: 100%; Specificity: 94%; PPV (1 pt.): 93.8%; PPV (2 pts.): 100%; NPV (0 pts.): 100%.	Internal validation: Boruta + Logistic Regression. Small, single-center (*n* = 33). Informal external validation.
Gao Nomogram [87]	iPTH (continuous); ALP (continuous).	Logit(P) = −0.253 + 0.0095 × ALP + 0.00105 × iPTH.	Continuous risk probability (0–100%).	C-index: 0.943 (0.892–0.994); AUC (iPTH): 0.873; AUC (ALP): 0.926; H-L: χ^2^ = 3.405, *p* = 0.474; Cutoffs: iPTH 2433.1 pg/mL, ALP 289.5 U/L.	Internal bootstrap validation (1000 iterations); Single center (*n* = 75); Excellent calibration.
Wang Nomogram [24]	Continuous values for: iPTH; BALP; Corrected Ca^2+^; Gland weight (g).	Visual Nomogram.	Continuous risk probability (0–100%).	Outperforms single predictors: Nomogram AUC ≈ 0.920 > 0.844 (iPTH); 0.776 (BALP); 0.701 (weight); 0.655 (Ca^2+^).	Internal bootstrap validation (1000 iterations); Single center (*n* = 114); HBS: 76.3%. No large external validation study until now.
USRDS Risk Score [98]	Age < 48 years (+2 pts.); Dialysis ≥ 5 years (+1 pt.); Renal osteodystrophy (+1 pt.); Kidney transplant vintage ≥3 years (+1 pt.); Elixhauser score ≥5 (+1 pt.).	Weighted β-coefficients: 0–6 pts.	0: ~8% risk; 1–2: 12–18%; 3–4: 26–35%; 5–6: ~44%.	Accuracy: 46.5%; Sensitivity: 75.6%; Specificity: 40.7%; PPV: 20.3%; NPV: 89.3%.	75% training, 25% validation; National registry (*n* = 17,074). HBS rates: from 8% (0 pts.) to 44% (6 pts.). ↑ ICU admissions in HBS: 33.5% vs. 24.6% (*p* < 0.001).
XGBoost ML [99]	Continuous values for: Age; Corrected Ca^2+^; ALP; Pre-PTX PTH; %PTH = PTH decay between Pre-PTX vs. at skin closure.	ML algorithm + SHAP	Low-risk: <20%; Moderate-risk: 20–35%; High-risk: ≥35%	AUC: 0.878 (0.779–0.973); F1 score: 0.871 (for the validation cohort); Sensitivity: 87.1%; Specificity: 81.8%.	70% training, 30% validation; Single center (*n* = 181); Factor selection: Logistic regression + Boruta algorithm. Available as Web application.

↑ = Increased/elevated; *n* = number of cases; HBS = hungry bone syndrome; PTX = parathyroidectomy; ALP = alkaline phosphatase; PTH/iPTH = (intact) parathyroid hormone; BALP = bone-specific alkaline phosphatase; Ca^2+^ = corrected calcium; NYU = New York University; USRDS = United States Renal Data System; XGBoost = Extreme Gradient Boosting; ML = machine-learning; SHAP = SHapley Additive exPlanations; AUC = Area Under Curve; PPV = Positive Predictive Value; NPV = Negative Predictive Value; H-L = Hosmer-Lemeshow test; ICU = intensive care unit; pt./pts. = point/points; Logit(P) = logistic transformation of probability. C-index is equivalent to AUC. Risk categories represent stratification groups for clinical decision-making. Validation methods: bootstrap resampling typically uses 1000 iterations; train-validation split indicates proportion of data used for model development versus testing.

## Abbreviations

The following abbreviations are used in this manuscript:

AAES	American Association of Endocrine Surgeons
ALP	Alkaline phosphatase
AUC	Area under curve
BALP	Bone-specific alkaline phosphatase
BMD	Bone mineral density
BTMs	Bone turnover markers
cAMP	Cyclic adenosine monophosphate
CaSR	Calcium-sensing receptor
CKD	Chronic kidney disease
CKD-MBD	Chronic kidney disease-mineral and bone disorder
CPTHR	C-terminal parathyroid hormone receptor
CTX	C-terminal telopeptide
EMR	Electronic medical record
ESRD	End-stage renal disease
FGF-23	Fibroblast growth factor-23
GFR	Glomerular filtration rate
HBS	Hungry bone syndrome
ICU	Intensive care unit
iPTH	Intact parathyroid hormone
IV	Intravenous
KDIGO	Kidney Disease: Improving Global Outcomes
KDOQI	Kidney Disease Outcomes Quality Initiative
LASSO	Least Absolute Shrinkage and Selection Operator
MCP-1	Monocyte chemoattractant protein-1
ML	Machine learning
MSCs	Mesenchymal stem cells
NPV	Negative predictive value
NTX	N-terminal telopeptide
NYU	New York University
OPG	Osteoprotegerin
OR	Odds ratio
P1NP	Procollagen type I N-terminal propeptide
PHPT	Primary hyperparathyroidism
PKA	Protein kinase A
PKC	Protein kinase C
PPV	Positive predictive value
PTH	Parathyroid hormone
PTH1R	Parathyroid hormone/parathyroid hormone-related peptide receptor type 1
PTHrP	Parathyroid hormone-related peptide
PTX	Parathyroidectomy
RANKL	Receptor activator of nuclear factor kappa-B ligand
ROC	Receiver operating characteristic
SHAP	SHapley Additive exPlanations
SHPT	Secondary hyperparathyroidism
TRAP-5b	Tartrate-resistant acid phosphatase 5b
USRDS	United States Renal Data System
VDRs	Vitamin D receptors
XGBoost	Extreme Gradient Boosting
